# A Skin‐Interfaced, Miniaturized Microfluidic Analysis and Delivery System for Colorimetric Measurements of Nutrients in Sweat and Supply of Vitamins Through the Skin

**DOI:** 10.1002/advs.202103331

**Published:** 2021-11-07

**Authors:** Joohee Kim, Yixin Wu, Haiwen Luan, Da Som Yang, Donghwi Cho, Sung Soo Kwak, Shanliangzi Liu, Hanjun Ryu, Roozbeh Ghaffari, John A. Rogers

**Affiliations:** ^1^ Center for Bio‐Integrated Electronics Northwestern University Evanston IL 60208 USA; ^2^ Querrey Simpson Institute for Bioelectronics Northwestern University Evanston IL 60208 USA; ^3^ Department of Materials Science and Engineering Northwestern University Evanston IL 60208 USA; ^4^ Department of Biomedical Engineering Northwestern University Evanston IL 60208 USA; ^5^ Center for Bionics of Biomedical Research Institute Korea Institute of Science and Technology Seoul 02792 Korea; ^6^ Department of Mechanical Engineering Northwestern University Evanston IL 60208 USA; ^7^ Department of Neurological Surgery Feinberg School of Medicine Northwestern University Chicago IL 60611 USA

**Keywords:** nutrient delivery, nutrition, skin‐interfaced systems, sweat sensors, wearable sensors

## Abstract

Nutrients play critical roles in maintaining core physiological functions and in preventing diseases. Technologies for delivering these nutrients and for monitoring their concentrations can help to ensure proper nutritional balance. Eccrine sweat is a potentially attractive class of biofluid for monitoring purposes due to the ability to capture sweat easily and noninvasively from nearly any region of the body using skin‐integrated microfluidic technologies. Here, a miniaturized system of this type is presented that allows simple, rapid colorimetric assessments of the concentrations of multiple essential nutrients in sweat, simultaneously and without any supporting electronics – vitamin C, calcium, zinc, and iron. A transdermal patch integrated directly with the microfluidics supports passive, sustained delivery of these species to the body throughout a period of wear. Comparisons of measurement results to those from traditional lab analysis methods demonstrate the accuracy and reliability of this platform. On‐body tests with human subjects reveal correlations between the time dynamics of concentrations of these nutrients in sweat and those of the corresponding concentrations in blood. Studies conducted before and after consuming certain foods and beverages highlight practical capabilities in monitoring nutritional balance, with strong potential to serve as a basis for guiding personalized dietary choices.

## Graphical abstract

This paper introduces a soft, skin‐interfaced microfluidic system that enables nutrient analysis in sweat and delivery of vitamins to the body. Accurate colorimetric sensors can quantify the concentrations of nutrients in sweat, and a transdermal patch integrated directly with the microfluidics supports passive, sustained delivery of nutrients to the body throughout a period of wear.

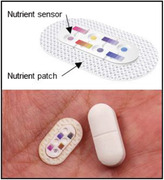

## 1 Introduction

Essential nutrients are necessary for basic physiological functions that underpin good health. Deficiencies can lead to myriad conditions, ranging from digestive complications, skin disorders, cardiac diseases, bone defects, and even dementia.^[^ ^1^, ^2^ ^]^ Lack of nutritional balance can create complications in disease recovery because consumption of vitamins and minerals (calcium, zinc, and iron) at adequate levels is critical for maintaining the immune system.^[^ ^3^, ^4^, ^5^ ^]^ One of the most serious concerns is that malnutrition in children can impede proper physical and mental development, with long‐lasting and often permanent effects. Nearly 50 distinct nutrients are known to participate at various levels in essential body processes.^[^ ^6^ ^]^ The work presented here focuses on several of the most important of these, including vitamin C, which is essential for healthy bones and blood vessels, for regenerative cascades in wound healing, and for mechanisms that govern the absorption of iron in hemoglobin. Deficiencies in vitamin C can lead to scurvy, a disorder characterized by hemorrhagic manifestations and abnormal osteoid and dentin formation.^[^ ^7^ ^]^ A second nutrient is iron, essential for a variety of metabolic processes including oxygen transport and deoxyribonucleic acid (DNA) synthesis. Deficiencies in iron lead to a broad spectrum of diseases, ranging from anemia to iron overload, and possibly to neurodegenerative diseases.^[^ ^8^ ^]^ Calcium is a third nutrient of interest due to its dominating presence as mineral content of bones and teeth.^[^ ^9^ ^]^ Lack of calcium can lead to dental changes and osteoporosis. A fourth key nutrient is zinc, a species involved in the catalytic activity of nearly 100 known enzymes, with relevance to immune‐system‐related diseases including malabsorption syndrome, chronic liver disease, chronic renal disease, and sickle cell disease.^[^ ^10^ ^]^ These and other nutrients participate in vital, often synergistic roles at every stage of the immune response, thus motivating the need for measurements of multiple species, simultaneously in a single wearable platform.^[^ ^11^ ^]^

Proper nutritional balance of these and other species follows naturally from a healthy diet. Vitamin fortification of certain types of foods and supplements in the form of tablets or skin‐interfaced patches can complement proper dietary selections. Assessments of nutrition typically rely on clinical and/or anthropometric measurements that follow strict protocols, typically performed or guided by a trained professional, to record not only the amount and type of food intake, but also many other factors. Differences in biochemistry, metabolism, genetics, and microbiota contribute to dramatic interindividual differences in the response of nutritional balance to dietary patterns, the timing of eating, and environmental exposure.^[^ ^12^ ^]^ These and other known effects create significant uncertainties in such types of assessments. The most accurate approaches involve direct screening of biomarkers in the blood,^[^ ^13^ ^]^ although typically conducted only in an episodic mode through blood draws performed in health clinics followed by lab analysis. An ideal alternative would exploit a similarly direct approach, but with classes of biofluid that can be accessed frequently and noninvasively, to allow monitoring of key nutrients as they vary across timescales from hours to days, weeks, and months. The resulting information could serve as immediate feedback to help guide healthy dietary choices and informed use of dietary supplements.

Among various biofluids that can be captured noninvasively, eccrine sweat is interesting because, unlike saliva and tears, it can be collected and analyzed in situ using soft microfluidic lab‐on‐a‐chip‐type devices mounted at nearly any location across the body, reproducibly and in pristine, small quantities. Sweat contains a multitude of biochemical markers that have been shown to correlate to physiological health status, including micronutrients, hormones, electrolytes, and metabolites.^[^ ^14^ ^]^ Previous work describes various classes of wearable sweat microfluidic sensors for continuous monitoring of metabolites (glucose,^[^ ^15^, ^16^, ^17^, ^18^ ^]^ lactate,^[^ ^15^, ^19^, ^20^ ^]^ cortisol,^[^ ^18^, ^21^ ^]^ urea,^[^ ^22^ ^]^ creatinine,^[^ ^22^ ^]^ and alcohol^[^ ^23^, ^24^ ^]^) and electrolytes (chloride,^[^ ^15^, ^20^, ^25^ ^]^ sodium,^[^ ^15^, ^26^ ^]^ potassium,^[^ ^15^, ^16^ ^]^ calcium,^[^ ^27^ ^]^ and zinc^[^ ^24^ ^]^) in sweat. Of particular relevance to nutritional monitoring, recent publications describe classes of electrochemical sensors of vitamin C in sweat deployed at the interface between the device and the surface of the skin.^[^ ^24^, ^28^, ^29^ ^]^ The results demonstrate an ability to track dynamic changes in the concentration of vitamin C in sweat following consumption of vitamin C supplements and beverages rich in vitamin C (vitamin C water, or orange juice).^[^ ^24^, ^28^, ^29^ ^]^ These emerging capabilities in sweat collection and analysis suggest opportunities for the development of simple, nonelectronic microfluidic technologies that can rapidly measure the nutritional status, with additional sensing of water loss through sweat to quantitatively inform fluid and nutrient replenishment strategies. In situ sensing approaches based on colorimetric, fluorometric, and related schemes have the potential to yield quantitative information on biomarker concentrations from volumes of sweat in the microliter range, or less. The vision is for a technology‐enabled, data driven approach to managing personal health, with minimal burden on the subject using miniaturized devices mounted on any convenient location of the anatomy. A system with the ability to both deliver and analyze nutrients could represent an additional useful level of function in maintaining proper nutritional balance and homeostasis.

Here, we present a soft, skin‐interfaced microfluidic system that offers these and other sensing and delivery capabilities, demonstrated in a simple, low cost, millimeter‐scale platform that allows monitoring and delivery of vitamin C, calcium, zinc, and iron, in nearly any setting, without the need for electronics or supporting hardware such as batteries and wireless communication systems. Several unique aspects support these modes of operation. First, simple colorimetric assays monitor nutrients in sweat that collects in separate microscale reservoirs following passage from the surface of the skin through a collection of microchannels and passive valving structures. Second, the small overall sizes of these devices and the large depths of their analysis reservoirs represent additional features that distinguish this technology over those in previous publications. As a further qualitative distinction, patches integrated with these platforms deliver nutrients transdermally via well‐established diffusive schemes, resulting in a single platform that offers the ability to both noninvasively deliver and analyze nutrients in sweat. The materials and fabrication approaches have the potential to support cost‐effective, high volume production, with broad applicability in many scenarios. Concentrations of nutrients and their dynamic variations following oral intake and transdermal delivery yield key information on nutritional balance. Quantitative comparisons of the time‐dependent concentrations of these species in sweat and in blood plasma suggest that sweat chemistry approximately correlates to blood chemistry in the cases examined. Examples in monitoring changes in the concentrations of nutrients in sweat after consuming various foods and beverages demonstrate the practical potential of these ideas. The results suggest that wearable sweat microfluidics systems with integrated colorimetrics could yield personalized data directly relevant to nutritional balance, with implications for decentralized access to care and improved health outcomes.

## 2 Results and Discussions

### 2.1 Miniaturized, Soft Epidermal Microfluidic Systems for Monitoring Nutrients and Delivering Vitamins

**Figure 1:**
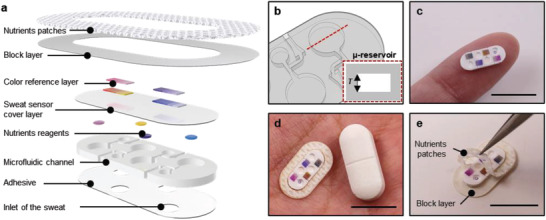
Schematic illustrations and optical images of a miniaturized sweat microfluidic device designed to measure four key sweat nutrients via colorimetric analysis, and to serve as a mounting location for a transdermal nutrient patch. a) Schematic illustration in exploded view of the design of the microfluidic sensor and the nutrient patch. b) Diagram of the geometry of a microreservoir for colorimetric analysis. The red dashed box highlights the depth of the structure. c) Image of the device mounted on the fingertip. Scale bar: 1 cm. d) Optical image of a complete system placed next to a vitamin tablet. Scale bar: 1 cm. e) Image of the nutrient patch. Scale bar: 1 cm.

As shown in the schematic illustrations of **Figure** **1**a, the device platform described here adopts the form of a soft, oval shape (10 mm × 5 mm) designed to capture and route sweat through a collection of passive microvalves, a network of microchannels, and a set of microreservoirs (µ‐reservoirs), each with a colorimetric chemical reagent for sensing the concentration of a key nutrient (vitamin C, calcium, zinc, and iron). An oval‐shaped medical grade adhesive (1524 skin adhesive, 3M, Inc.; thickness 60 µm) with openings that define areas for collecting sweat as it emerges from the surface of the skin (corresponding to ≈10 sweat glands)^[^ ^30^ ^]^ establishes a water‐tight seal and a stable, strong adhesive interface between the base of the device and the surface of the skin. The overall construction relies on low‐modulus elastomeric materials, patterned and molded using the techniques soft lithography and laser ablation. The specific layout includes four repeated sets of microchannels and µ‐reservoirs for measurements of concentrations of vitamin C, calcium, zinc, and iron in sweat. Each set consists of a circular µ‐reservoir that fills with sweat (Figure S1, Supporting Information) due to the combined action of pressures created by eccrine sweat glands (≈70 kPa)^[^ ^31^ ^]^ and of passive, capillary bursting valves (CBVs) integrated into the microchannel network.^[^ ^32^ ^]^ These CBVs prevent excess sweat from interfering with sensing processes in the reservoirs by routing this sweat through connecting microfluidic channels and outlet ports (Figure S1c,d, Supporting Information).

These microfluidic design features appear in other, previously reported devices for sweat collection and analysis. An important additional aspect of the devices reported here enables enhanced measurements from the colorimetric assays through the use of µ‐reservoirs with thicknesses (≈1000 µm), and therefore optical path lengths for reflection mode readout, that are comparatively larger than those in other platforms (Figure 1b and Figure S2 (Supporting Information)). Careful choices of layout and constituent materials allow for thicknesses in this range, without mechanical collapse of the microchannels or µ‐reservoirs^[^ ^33^ ^]^ and without significantly compromising the soft, flexible mechanics needed for comfortable interfaces to the skin. The base layer of the device consists of polydimethylsiloxane (PDMS; Sylgard 184, Dow Corning) doped with a white pigment (10% by weight), and in a structure with relief features that define the microfluidic layout. A transparent capping layer of PDMS (thickness 200 µm) seals the system and serves as a support for a thin transparent polyester adhesive film (THERML film SELECT 10852; FLEXcon, MA, USA; thickness 25 µm). Color reference markers printed onto this patterned film aid in the quantitative extraction of color information from digital images of the device, with little dependence on the color temperature of the ambient lighting.^[^ ^32^ ^]^ Figure 1c shows an image of a representative miniature sweat microfluidic device on a fingertip.

Patches that contain relevant vitamins release these species to the surface of the skin, where they passively diffuse into the capillary beds in the dermis and subsequently throughout the bloodstream with kinetics that depend on the permeability of the stratum corneum and the concentration. These patches also include various chemical enhancers to increase skin permeability by altering the lipid structures of the stratum corneum.^[^ ^34^, ^35^ ^]^ The nutrient patch mounts around the perimeter of the device, as shown in Figure 1d. The overall size of the integrated system is similar that of a vitamin tablet. In one use case, the device can visually alert a user to an insufficient nutrient balance, thereby prompting him/her to remove the blocking layer of the nutrient patch to initiate the release process (Figure 1e). Additional details of the fabrication and integration strategies are in Figure S3 (Supporting Information).

### 2.2 Quantitative Colorimetric Assays of Vitamin C, Calcium, Zinc, and Iron

Analyte solutions with well‐defined concentrations of target species serve as the basis for assessing the performance of each assay. The vitamin C assay relies on the ferric reducing/antioxidant and ascorbic acid (FRASC) system.^[^ ^36^ ^]^ Here, ferric ion (Fe^3+^) reduces to ferrous ion (Fe^2+^) through interactions with vitamin C, to generate a compound with a pink color. Drop‐casting the FRASC solution into the µ‐reservoirs and drying under vacuum creates films that can be activated upon interaction with sweat. Similar casting schemes yield assays for calcium, zinc, and iron. Calcium sensing follows from the reaction of calcium with *o*‐cresolphthalein complexone (*o*‐CPC), to yield a violet‐colored complex.^[^ ^37^ ^]^ The zinc sensor involves a 2‐(5‐bromo‐2‐pyridylazo)‐5‐[*N*‐propyl‐*N*‐(3‐sulfopropyl)‐amino]‐phenol (5‐Br‐PAPs) as a chelating agent that binds zinc with high affinity, leading to a color change from yellow to pink.^[^ ^38^ ^]^ Finally, iron relies on 3‐(2‐pyridyl)‐5,6‐difurylsulfonic acid‐1,2,4‐triazine disodium salt (Ferene‐S) to produce a blue complex.^[^ ^39^ ^]^ In all cases, the assays build on kits originally designed for use with serum, urine, saliva, and other biological fluids, with high selectivity to targeted species contained in these biofluids and also in sweat. Although chromium is known to interfere with Ferene‐S, sweat contains 10 times more iron than chromium and thus can generally be ignored.^[^ ^40^ ^]^ Additional experimental details are in the Experimental Section part.

**Figure 2:**
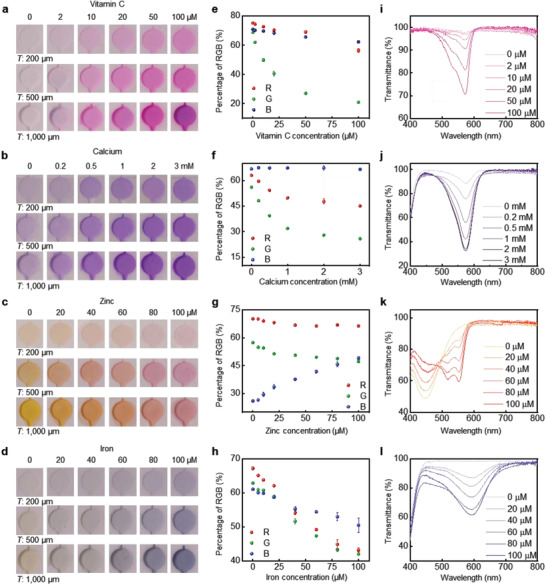
Quantitative colorimetric analysis of the responses of the nutrient assays by digital image processing and ultraviolet (UV)–visible spectroscopy. Optical images of µ‐reservoirs with different depths at various concentrations of a) vitamin C, b) calcium, c) zinc, and d) iron. Standard calibration curve between the normalized percentage of red, green, and blue levels from RGB analysis of the image and the concentration of e) vitamin C, f) calcium, g) zinc, and h) iron. Each data point in (e–h) indicates the average of measurements for three samples, and the error bars represent the standard deviations. The measured transmittance at various concentrations of i) vitamin C, j) calcium, k) zinc, and l) iron.

A typical healthy person releases sweat that contains 1 × 10^−6^–37 × 10^−6^ m vitamin C,^[^ ^28^ ^]^ 0.2 × 10^−3^–11 × 10^−3^ m calcium,^[^ ^41^ ^]^ 5 × 10^−6^–17 × 10^−6^ m zinc,^[^ ^40^, ^42^ ^]^ and 0.4 × 10^−6^–20 × 10^−6^ m iron.^[^ ^43^, ^44^ ^]^ **Figure** **2**a–d shows the color responses of the vitamin C, calcium, zinc, and iron sensors across these ranges of concentration, for µ‐reservoirs with different thicknesses. In all cases for pH values close to those of sweat and for physiological temperatures, the color responses stabilize in <5 min (Figure S4, Supporting Information). As expected, the strength of the color response increases with optical path length and, therefore, with depth of the µ‐reservoirs (200, 500, and 1000 µm). RGB values extracted from digital images under white light conditions indicate that the green level decreases with increasing concentration (Figure 2e–h). The sensitivity, as defined by the percentage change in the *G* value with concentration, for vitamin C, calcium, zinc, and iron is 0.46% µm ^−1^, 10.06% mm ^−1^, 0.16% µm ^−1^, and 0.21% µm ^−1^, respectively. The sensitivity increases by ≈2 times with an increase in the depth of the µ‐reservoir from 200 to 1000 µm, as exhibited in Figure S5 (Supporting Information). Measurements by ultraviolet (UV)–visible spectroscopy (Figure 2i–l) show similar trends. Here, the molar absorptivity (*ε*) can be calculated using the UV–visible spectroscopy results and Beer's law (Equation (1)). The absorbance (*A*) depends linearly on the analyte concentration (*c*)

(1) A=ε·l·c

where *ε* is the molar absorptivity of analyte, and *l* is the length of sample through which light passes from the source. As illustrated in Figure S6 (Supporting Information), the slope of the linear fit (absorbance as a function of concentration) equals the product of the molar absorptivity coefficient and the optical path length (*εl*). From the experimental results, the molar absorptivity (*ε*) for vitamin C, calcium, zinc, and iron is 15 200, 1447, 20 290, and 20 460 L mol^−1^ cm^−1^, respectively. Increasing the thickness from 200 to 1000 µm decreases the percent transmission by ≈2 times. The experimentally measured *G* value also increases significantly, as expected.

Additional experiments define the dependence of these assays on pH and temperature. Sweat is normally slightly acidic, with pH typically between 4.5 and 7.0.^[^ ^45^, ^46^ ^]^ Results of tests in buffer solutions with pH 4.5, 5.5, 6.5, and 7, as shown in Figure S7 (Supporting Information), indicate that the responses of the colorimetric assays change only slightly (decrease of the sensitivity 0.1% µm ^−1^ for vitamin C, 0.83% mm ^−1^ for calcium, 0.02% µm ^−1^ for zinc, and 0.06% µm ^−1^ for iron) over this range. The temperature of the sweat, enclosed in devices that are in intimate contact with the skin, will be comparable to that of the body, between 33.5 and 36.9 °C.^[^ ^47^ ^]^ Results obtained across a broad range, from 20 to 50 °C (Figure S8, Supporting Information), reveal negligible dependence of the response on temperature (decrease of the sensitivity 0.02% µm ^−1^ for vitamin C, 1.30% mm ^−1^ for calcium, 0.003% µm ^−1^ for zinc, and 0.05% µm ^−1^ for iron).

### 2.3 Transdermal Delivery of Nutrients and On‐Body Measurement of Sweat Vitamin C, Calcium, Zinc, and Iron

**Figure 3:**
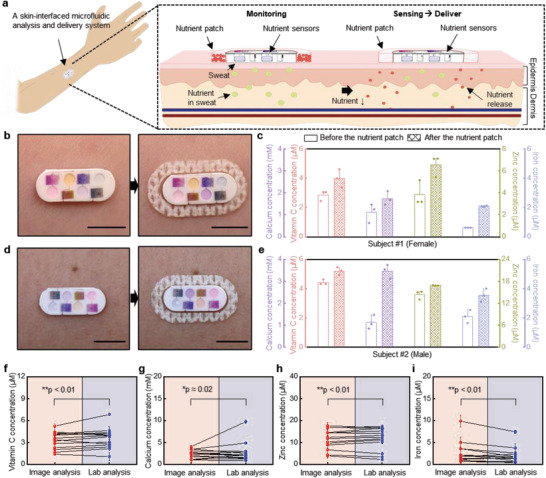
Human trials of devices for assessment of nutrient concentrations in sweat before and after applying the nutrient patches. a) Schematic illustration of the approach to monitor concentrations of nutrients in sweat and to deliver nutrients using a transdermal patch. b) Image of a device with a nutrient patch captured before and after sweating with subject #1. Scale bars: 0.5 cm. c) The concentration nutrients in the sweat of subject #1 before and after applying patch for 4 h. d) Image of a device with a nutrient patch captured before and after sweating with subject #2. Scale bars: 0.5 cm. e) The concentration nutrients in the sweat of subject #2 before and after applying patch for 4 h. Each data point in (c, e) indicates the average value calculated from three measurements with each device. Error bars correspond to standard deviations. The concentrations of f) vitamin C, g) calcium, h) zinc, and i) iron in sweat obtained by image processing of data from the device versus lab analysis of sweat (*n* = 7). *p < 0.5, **p < 0.1, two‐tailed test.

The design concept includes a transdermal patch that delivers nutrients to the body with sweat microfluidic sensors that monitor the uptake of these nutrients, as a system for proper nutrition management (**Figure** **3**a). Experiments on sweat chemistry evaluated before and after applying a patch with 2500 mg of vitamin C, 1400 mg of calcium, 30 mg of zinc, and 50 mg of iron reveal time‐dependent variations in concentrations that can be compared to those previously studied for similar nutrients in blood. The studies summarized in Figure 3 involve healthy, consented human volunteers (*n* = 4 males and *n* = 3 females) with devices mounted on various, thoroughly cleaned body locations, during sweating induced by sessions in a sauna room (Figure S9, Supporting Information). Analysis of digital images at three different locations from each reservoir yields averaged color readings that can be correlated to quantitative values of concentration for each nutrient using an appropriate calibration factor (Figure S10, Supporting Information).

Figure 3b–e shows devices before and after applying the nutrient patch, along with data acquired from the sensors for subjects #1 and #2, respectively. Figure S11 (Supporting Information) shows results for the other subjects (Subjects #3–#7). Nutrition concentrations before applying the patch are 1.37 × 10^−6^–4.40 × 10^−6^ m for vitamin C, 0.93 × 10^−3^–3.4 × 10^−3^ m for calcium, 3.84 × 10^−6^–14.46 × 10^−6^ m for zinc, and 0.63 × 10^−6^–4.98 × 10^−6^ m for iron. Most of these values are within the expected physiological ranges for healthy individuals.^[^ ^28^, ^41^, ^42^, ^43^, ^44^ ^]^ Data from seven volunteers with patches mounted on the skin for 4 h, corresponding to a duration that is approximately one half of the maximum sustained release reported in the specifications document for the patch, reveal that the nutrient concentrations in sweat increase to 0.79 × 10^−6^ ± 0.20 × 10^−6^ m for vitamin C, 0.82 × 10^−3^ ± 0.68 × 10^−3^ m for calcium, 2.99 × 10^−6^ ± 1.04 × 10^−6^ m for zinc, and 1.80 × 10^−6^ ± 1.34 × 10^−6^ m for iron. Although the magnitudes of these changes vary with subject, all seven subjects show significant increases.

Studies that use absorbing pads applied onto the skin and laboratory‐based analysis based on inductively coupled plasma mass spectrometry (ICP‐MS) and spectrophotometer provide comparative data. For these experiments, nutrient concentrations measured before and after applying the patches onto seven different subjects yield a total of 14 data points for comparison. As shown in Figure 3f–i, concentrations of the nutrients (vitamin C, calcium, zinc, and iron) obtained by colorimetric readouts performed on the microfluidic devices while on the skin show excellent agreement with these laboratory measurements. These two different methods for measuring the concentrations of these nutrients show a strong Pearson correlation (0.926 for vitamin C, 0.743 for calcium, 0.895 for zinc, and 0.963 for iron) with the low two‐tailed *p* value (**p < 0.01 for vitamin C, *p ≈ 0.02 for calcium, **p < 0.01 for zinc, and **p < 0.01 for iron).

### 2.4 Comparisons of Sweat Vitamin C, Calcium, Zinc, and Iron Following Transdermal and Oral Nutrient Delivery

**Figure 4:**
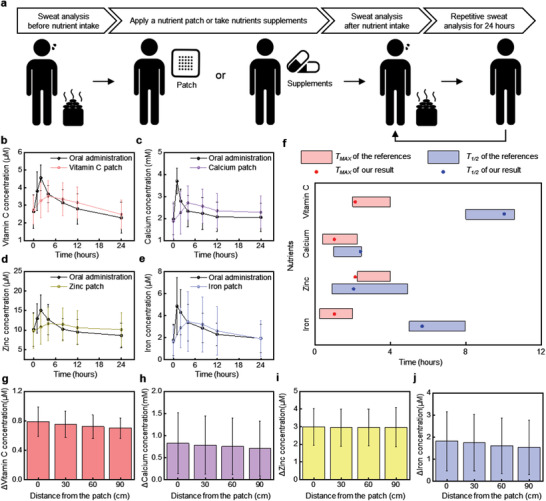
Comparison of the concentrations of nutrients in sweat across seven subjects following delivery of nutrients via a transdermal patch and multivitamin tablet. a) Cartoon diagram of the experimental protocols. Comparison of the time variation of concentrations of b) vitamin C, c) calcium, d) zinc, and e) iron in sweat at different time intervals after application of transdermal nutrient patches and oral administration of multivitamin tablets. All values are mean ± SD (*n* = 7). f) Comparison of *T*_Max_ and *T*_1/2_ between our results for and previous reported results for plasma. The relative change of concentrations of g) vitamin C, h) calcium, i) zinc, and j) iron in sweat at various distances from the nutrient patch. Each subject was measured with a single device.

A key advantage of transdermal delivery of nutrients using patch‐type platforms over oral consumption of vitamin tablets is in a gradual, continuous release over long periods of time.^[^ ^48^ ^]^ Evaluations with human subjects focus on monitoring the concentrations of sweat nutrients over 24 h after application of a nutrient patch and after oral administration of a multivitamin supplement. As illustrated in **Figure** **4**a, the experiments begin with collection of sweat from seven subjects during sessions in a sauna, before nutrient intake. The subjects then mount a nutrient patch on the chest, arm, or back. On another day, these same participants take nutrient supplements orally. Both transdermal and oral administration begin at around 8 a.m. Measurements of the concentrations of nutrients in sweat then occur at 1, 2, 4, 8, 12, and 24 h after administration, again during a session in a sauna. The data in Figure 4b–e compare the time course of results for vitamin C, calcium, zinc, and iron, for both oral and transdermal cases. The administration of each of these nutrients through the transdermal route results in a comparatively constant concentration in sweat, with reduced probability of abrupt increases in relative to oral delivery. For transdermal administration, initially low baseline concentrations (vitamin C: 2.91 × 10^−6^ ± 1.00 × 10^−6^ m, calcium: 1.89 × 10^−3^ ± 0.86 × 10^−3^ m, zinc: 9.78 × 10^−6^ ± 4.43 × 10^−6^ m, and iron: 1.64 × 10^−6^ ± 1.44 × 10^−6^ m) quickly increase by 1.3 times for vitamin C, 1.4 times for calcium, 1.2 times for zinc, and 2.1 times for iron, over 4 h after applying the patch. At 8 h, the concentrations reach levels below their maximum but they remain higher than concentrations at the same time point after oral administration. In the oral case, peak concentrations are larger than baseline levels by 1.73 times for vitamin C, 1.87 times for calcium, 1.48 times for zinc, and 2.73 times for iron. These behaviors are similar to previously published pharmacokinetic profiles of concentrations in plasma after oral dosage.^[^ ^49^, ^50^, ^51^, ^52^ ^]^

Quantitative comparisons of the dynamic concentrations in sweat and in plasma involve measurements of the time required to reach the maximum concentrations (*T*_Max_) and the time (*T*_1/2_) required to decrease to one half of these maximum concentrations. As shown in Figure 4f, *T*_Max_ and *T*_1/2_ for sweat are similar to those for plasma in all cases. These results suggest that sweat chemistry correlates, at least semiquantitatively, to plasma chemistry for these species, in the contexts described here. The microfluidic architecture of these devices allows additional studies of the effects of sweat rates and methods for sweat induction, to be examined in future work.

Additional human subject tests explore the spatial extent of diffusion of nutrients through the body, from the patch. These experiments focus on the corresponding concentrations in sweat before and after applying the nutrient patch on the chest for 4 h, at a distance of 30 cm (around wrist), 60 cm (around thigh), and 90 cm (around leg). As shown in Figure 4g–j, the results indicate that the concentrations vary only slightly with distance (vitamin C: 0.09 × 10^−6^ m, calcium: 0.12 × 10^−3^ m, zinc: 0.05 × 10^−6^ m, and iron: 0.28 × 10^−6^ m), in all cases remain higher after than before applying the patch. This behavior is consistent with rapid, local diffusion of nutrients into the bloodstream, followed by flow‐induced transport across the body via blood circulation.

### 2.5 On‐Body Measurements of Sweat Vitamin C, Calcium, Zinc, and Iron after Consumption of Food and Drink during Daily Living

**Figure 5:**
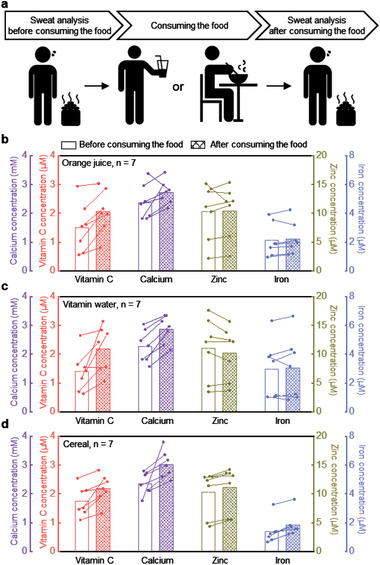
On‐body evaluation of changes in concentrations of nutrients in sweat associated with consumption of orange juice, vitamin water, and cereal. a) Schematic illustration of the experiments. Changes in concentrations before and b) after drinking the orange juice, c) after drinking the vitamin water, d) after consuming cereal (*n* = 7). Each subject was measured with a single device.

The use of the wearable technology introduced here is most powerful in tracking nutrients for different relevant dietary scenarios in daily living. On‐body evaluations of this capability involve monitoring changes in the concentrations of nutrients in the sweat after consuming orange juice, vitamin water, and cereal, each with different nutrient components (Table S1, Supporting Information). As illustrated in **Figure** **5**a, the study protocols rely on measurements across seven human subjects before and after consumption. Figure 5b shows the changes in nutrient levels after drinking 300 mL of orange juice, corresponding to an intake of ≈63 mg of vitamin C and 20 mg of calcium. The concentrations of vitamin C and calcium exhibit notable increases (change of the vitamin C concentration: 0.56 × 10^−6^ ± 0.31 × 10^−6^ m, change of the calcium concentration: 0.35 × 10^−3^ ± 0.47 × 10^−3^ m), qualitatively consistent with expectation. Changes in zinc and iron are comparatively small (change of the zinc concentration: 0.12 × 10^−6^ ± 1.36 × 10^−6^ m, change of the iron concentration: 0.08 × 10^−6^ ± 0.19 × 10^−6^ m), consistent with the relative absence of these species in orange juice. Vitamin water (1 bottle, 591 mL) includes 135 mg of vitamin C and 80 mg of calcium. As a consequence, as shown in Figure 5c, the concentrations of vitamin C and calcium in sweat exhibit larger changes than those associated with orange juice (change of the vitamin C concentration: 0.78 × 10^−6^ ± 0.63 × 10^−6^ m, change of the calcium concentration: 0.59 × 10^−3^ ± 0.19 × 10^−3^ m). Like orange juice, vitamin water also does not contain iron and zinc; changes in the concentration of iron and zinc in sweat are negligible (change of the zinc concentration: −0.77 × 10^−6^ ± 0.97 × 10^−6^ m, change of the iron concentration: 0.07 × 10^−6^ ± 0.39 × 10^−6^ m). On the other hand, vitamin fortified cereal contains all of the nutrients (7.2 mg of vitamin C, 80 mg of calcium, 1.65 mg of zinc, and 10.8 mg of iron) that can be measured by the devices. Figure 5d shows that concentrations of vitamin C, calcium, zinc, and iron in sweat increase (change of the vitamin C concentration: 0.44 × 10^−6^ ± 0.30 × 10^−6^ m, change of the calcium concentration: 0.66 × 10^−3^ ± 0.57 × 10^−3^ m, change of the zinc concentration: 0.79 × 10^−6^ ± 0.37 × 10^−6^ m, and change of the iron concentration: 0.43 × 10^−6^ ± 0.19 × 10^−6^ m) following consumption of cereal. However, the vitamin C content in cereal is smaller than that in orange juice or vitamin water, such that changes in vitamin C concentration are lower. Although the change in concentration of each nutrient in sweat does not exhibit a simple, linear relationship with intake, the general trend is an increase as with intake. These results represent promising indications of the ability to assess personalized nutrition information noninvasively, by analyzing sweat in a wearable microfluidic platform. A unique opportunity is in the deployment of this technology for personalized tracking over time, to gain insight individual into variability associated with diet, sleep, stress, metabolic rate, body type, ethnicity, gender, and other factors.

## 3 Conclusion

The miniaturized wearable microfluidic system introduced in this study allows routine monitoring of multiple nutrient components in sweat, and it provides a simple integrated capability for transdermal delivery of these and other nutrients. Demonstrations involve a microfluidic architecture and colorimetric scheme for rapid and quantitative analysis of vitamin C, calcium, zinc, and iron in sweat before and after transdermal delivery or oral administration of these and other species. The time dynamic concentrations of these species approximately follow those of blood, suggesting some value as correlative assessments that can be performed easily and rapidly by any user in any setting. The result of pilot studies indicate that different subjects exhibit different concentrations and temporal profiles of nutrients in sweat, thereby highlighting the need for personalized nutrition monitoring. The additional information on sweat rate and total sweat loss that follows from the use of microfluidics provides further value in managing personal health. In addition to the effects of sweat rate and total loss, additional opportunities are in the development of colorimetric chemistries based on enzymatic or DNA reactions. Other future directions are in improved approaches for nutrient delivery, including those that rely on active mechanisms, perhaps enhanced or triggered by sweat. The addition of electronic functionality could be relevant in this context, and as a means to extend the options in sensing. Electronic or pharmacological methods to stimulate the sweat glands are also of interest, to allow monitoring of nutritional status at particular time points, unconstrained by the need to engage natural mechanisms for sweat generation. This platform can be easily expanded to include other nutrients such as copper and certain amino acids through suitable modifications of established assay kits, thus establishing the basis for comprehensive nutrition panel on your skin. Future studies will examine the dependence of sweat rate and sweat loss on these and other species. The simplicity of these wearable devices, their capabilities in real‐time colorimetric readout, and their applicability in home settings suggest broad potential for a data driven, personalized approach to healthy nutritional intake.

## 4 Experimental Section

### Fabrication of the Microfluidic Channel

A thin layer of photoresist (KMPR 1010; MicroChem, MA, USA) spin‐cast (2000 rpm for 30 s) on a silicon wafer (2 mm thick) and then photolithographically patterned defined the geometry of the microfluidic channel and reservoirs. Subsequently, deep reactive ion etching (STS Pegasus ICP‐DRIE, SPTS Technologies Ltd.) created trenches with depths of 200, 500, and 1000 µm on the surface of the silicon wafer. Next, a spin‐cast layer of poly(methylmethacrylate) (PMMA; Microchem, MA, USA; 3000 rpm for 30 s) served as an antiadhesion layer. After fabrication of this mold, a mixture of a white dye (Reynolds Advanced Material, IL, USA, 5 wt%) and a precursor of PDMS (10:1 ratio of base to curing agent; Sylgard 184, Dow Corning, MI, USA) was spin‐cast at 200 rpm for 30 s and baked at 70 °C for 1 h to yield a soft, white microfluidic structure. A mechanical punch tool defined 0.3 mm diameter inlet holes for the microfluidic system. Assays for each of these nutrients were placed in the microfluidic reservoirs. Separately, spin casting PDMS (10:1) on a PMMA‐coated silicon wafer, then baking at 70 °C for 1 h yielded a 200 µm layer to seal the surface of the microfluidic structure. Last, a set of color reference markers printed on a thin transparent polyester adhesive film (THERML film SELECT 10852; FLEXcon, MA, USA; thickness 25 µm) using the commercial laser printer (Konica Minolta C454 PS Color, Tokyo, Japan) was bonded on top. Final assembly involved placing the nutrient patch in the side of the system. A corona treatment process enabled bonding of the bottom adhesive layer, microfluidic channel, cover layer, and color reference marker.

### Development of Colorimetric Assays for Nutrients

For colorimetric analysis, chromogenic reagents were formed as described in the following text, then drop cast into the reservoirs and dried in a vacuum chamber.

1) Vitamin C: The materials were purchased from Sigma‐Aldrich (MO, USA). The solution consisted of 1 µL of iron chloride solution and 1 µL of color probe dispersed in 8 µL buffer solution. A volume of 3 µL of this solution was cast and dried in a corresponding reservoir.
2) Calcium: The materials were purchased from Abcam (Cambridge, UK). The solution consisted of 18 µL of *o*‐CPC reagent dispersed in 12 µL of the buffer solution. A volume of 3 µL of this solution was cast and dried in a corresponding reservoir, repeated a total of 3 times.
3) Zinc: The materials were purchased from Abcam (Cambridge, UK). The solution was prepared by mixing a 5‐Br‐PAPs solution and a solution of salicylaldoxime at a ratio of 4:1 volume. A volume of 3 µL of this solution was cast and dried in a corresponding reservoir, repeated a total of 3 times.
4) Iron: The material was purchased from Abcam (Cambridge, UK). A volume of 3 µL of Ferene‐S solution was cast and dried in a corresponding reservoir.

### Preparation of Color Reference Markers

Color reference markers were prepared by exposure of each assay to standard nutrients and artificial sweat solutions, followed by capture of images of the assays using a digital camera (EOS 6D, Canon, Tokyo, Japan). Subsequently, images were analyzed by the program (Photoshop, Adobe Systems, CA, USA). A color laser printer produced a reference marker on a polyester adhesive film at 1200 dpi resolution. After placing the color reference markers adjacent to the microfluidic chambers, images of the devices were captured using a digital camera. The image analysis was performed as described in Figure S10 (Supporting Information).

### Preparation of Nutrient Patches

Patches that released vitamin C, calcium, zinc, and iron were purchased from PatchAid (NY, USA). A CO_2_ laser (Universal Laser Systems, AZ, USA) cut the patches into geometries to fit the side of the microfluidic system.

### Optical Characterization

The transmittance of the samples was measured by ultraviolet–visible–near infrared spectroscopy (PerkinElmer LAMBDA 1050, MA, USA). For this characterization, a transparent microfluidic channel was used instead of a white microfluidic channel.

### Measurements of Sweat Nutrient Level in Human Field Studies

Testing involved seven healthy adults (aged 20–35) as volunteers. All subjects provided their consent prior to participation. Prior to sweat monitoring, the skin was cleaned with an alcohol gauze pad, followed by attachment of the microfluidic systems on the desired body location. Each volunteer rested in a sauna up to 50 min. Digital images were collected for further analysis.

For the test in Figure 3, sweat samples were first monitored from each of the subjects, immediately prior to application of nutrient patches onto the skin. Sweat samples were collected again after 4 h. The trials in Figure 4 were conducted over two days. Evaluations focused on monitoring the concentrations of sweat nutrients over 24 h after application of a nutrient patch and after oral administration of a multivitamin supplement. Measurements of the concentrations of nutrients in sweat then occurred at 1, 2, 4, 8, 12, and 24 h after administration. For the sweat tests in Figure 5, the first sweat samples were monitored prior to consumption of cereal, orange juice, and vitamin water. Sweat samples were collected again after 2 h. The sensors were used once for each test.

### Lab‐Based Sweat Analysis

Sweat volumes of 30 µL were obtained from human subjects. Analysis of vitamin C relied on measurements of absorbance on 2 µL volumes of sweat placed on an optical probe operating at a wavelength of 570 nm (NanoDrop 2000, Thermo Fisher Scientific, MA, USA). For analysis of calcium, zinc, and iron, these samples were diluted into 3 mL 3% HNO_3_, without the need for additional heating because of the absence of organic chelates to form with the metals. Diluted samples were analyzed by ICP‐MS (Thermo iCap7600, Thermo Fisher Scientific, MA, USA).

### Statistical Analysis

Average values and error bars were calculated using Excel (Microsoft). Each data point in Figure 2e–h indicated the average of measurements with three devices, and the error bars represented the standard deviations (SDs). Each data point in Figure 3 indicated the average value calculated from three measurements for each device. Error bars corresponded to standard deviations. Pearson correlation analysis with two‐tailed *p* values was conducted using the SPSS statistical software (IBM, NY, USA) for Figure 3f–i. For Figures 4 and 5, all subjects were each measured with a single device, and each data point indicated the mean ± standard deviation for all subjects (*n* = 7).

## Conflict of Interest

The authors declare no conflict of interest.

## Supporting information

Supporting Information

## Data Availability

Research data are not shared.
