# Supplementary material for: A Skin‐Interfaced, Miniaturized Microfluidic Analysis and Delivery System for Colorimetric Measurements of Nutrients in Sweat and Supply of Vitamins Through the Skin
Source: Adv Sci (Weinh). 2021 Nov 7;9(2):2103331. doi: 10.1002/advs.202103331 (PMC8805554; doi:10.1002/advs.202103331)
Supplement: Supplementary file 1 — Supporting Information [file ADVS-9-2103331-s001.pdf]

WILEY-VCH

© 2021 Wiley-VCH GmbH

ADVANCED SCIENCE
Open Access

Supporting Information

for *Adv. Sci.,* DOI: 10.1002/advs.202103331

A skin-interfaced, miniaturized microfluidic analysis and delivery system for colorimetric measurements of nutrients in sweat and supply of vitamins

*Joohee Kim, Yixin Wu, Haiwen Luan, Da Som Yang, Donghwi Cho, Sung Soo Kwak, Shanliangzi Liu, Hanjun Ryu, Roozbeh Ghaffari, and John A. Rogers**

Supporting Information

A skin-interfaced, miniaturized microfluidic analysis and delivery system for colorimetric measurements of nutrients in sweat and supply of vitamins

*Joohee Kim, Yixin Wu, Haiwen Luan, Da Som Yang, Donghwi Cho, Sung Soo Kwak, Shanliangzi Liu, Hanjun Ryu, Roozbeh Ghaffari, John A. Rogers**

This section includes the following:

1. Supporting Information Text
2. Figure S1-S11
3. Table S1

Supporting Information Text

Calculation of bursting pressure of capillary bursting valves (CBVs)

The bursting pressure was calculated based on Young–Laplace equation.[32] The Young–Laplace equation gives the bursting pressure in a rectangular channel as

$$\text{Bursting pressure} = -2\sigma[\frac{\cos\theta_I^*}{b} + \frac{\cos\theta_A}{h}] \quad (1)$$

where σ is the surface tension of liquid, θ_A is the contact angle of the channel, θ_I* is the min [θ_A + β; 180°], β is the diverging angle of the channel, b and h are the width and the height of the diverging section, respectively. The CBVs #1, and #2 have diverging angles of 13° and 90°, respectively, and widths of 300 μm. The heights of the valves are 1,000 μm. PDMS, which is naturally hydrophobic, becomes hydrophilic after exposure to oxygen plasma for the purpose of activating the surfaces to enable bonding. The hydrophobicity recovers after ≈ 24 h, to reach a constant, time-independent contact angle of 107°.[32] Based on this parameter, the calculated bursting pressures for CBVs #1, and #2 are 0.29 kPa, and 0.52 kPa, respectively.”

Figures and Table

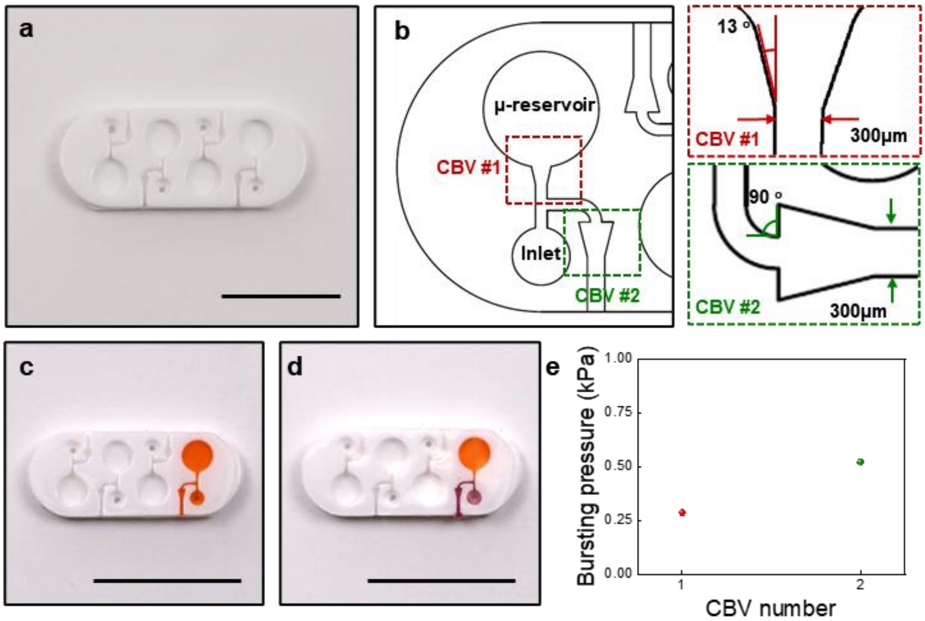

Figure S1. Capillary bursting valves (CBVs) designs for sequential sampling of sweat. a, Optical image of the CBVs. Scale bar, 1 cm. **b,** Detailed schematic illustration of sampling inlet, μ-reservoir, and the CBVs with different channel widths and diverging angles. Optical images of the mechanisms by which the action of the CBVs lead to filling **c,** μ-reservoir, **d,** microfluidic channel. Scale bars: 1 cm. **e,** Calculated bursting pressures of each capillary bursting valve.

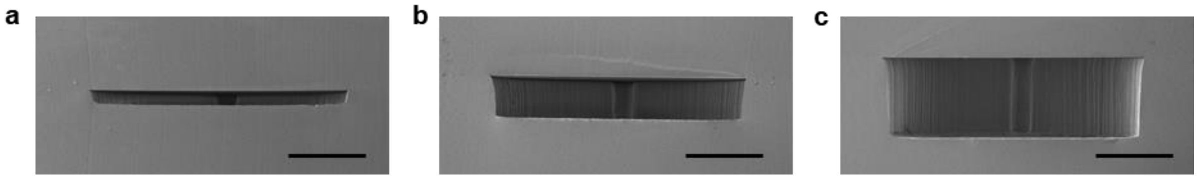

Figure S2. Cross-sectional scanning electron microscopy (SEM) images of microfluidic reservoirs at thicknesses of **a,** 200 µm, **b,** 500 µm, **c,** 1,000 µm. Scale bars: 1 mm.

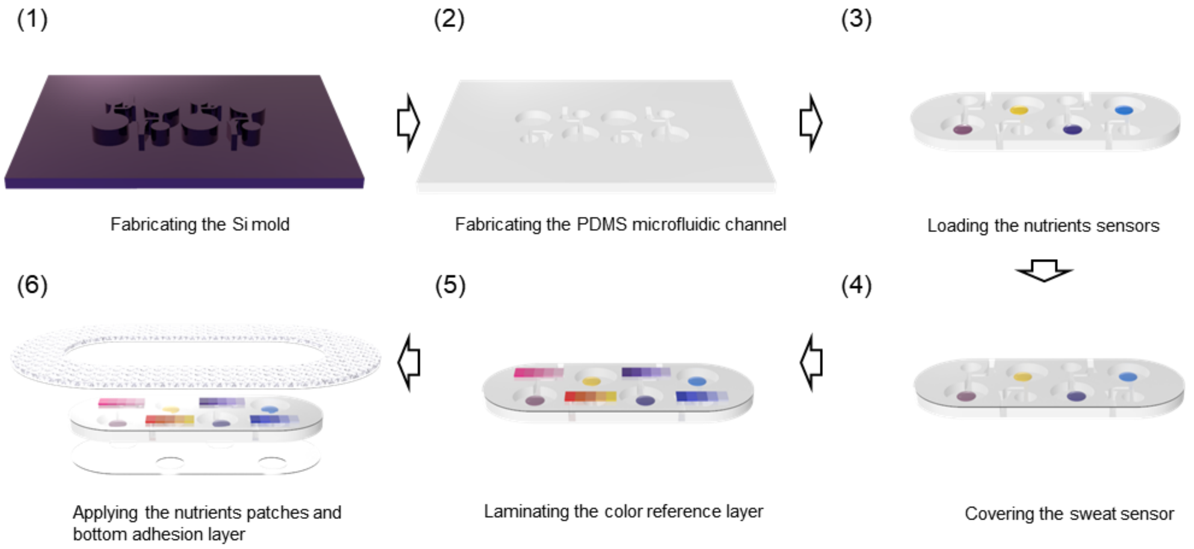

Figure S3. Schematic illustration of the fabrication procedures for the epidermal microfluidic device.

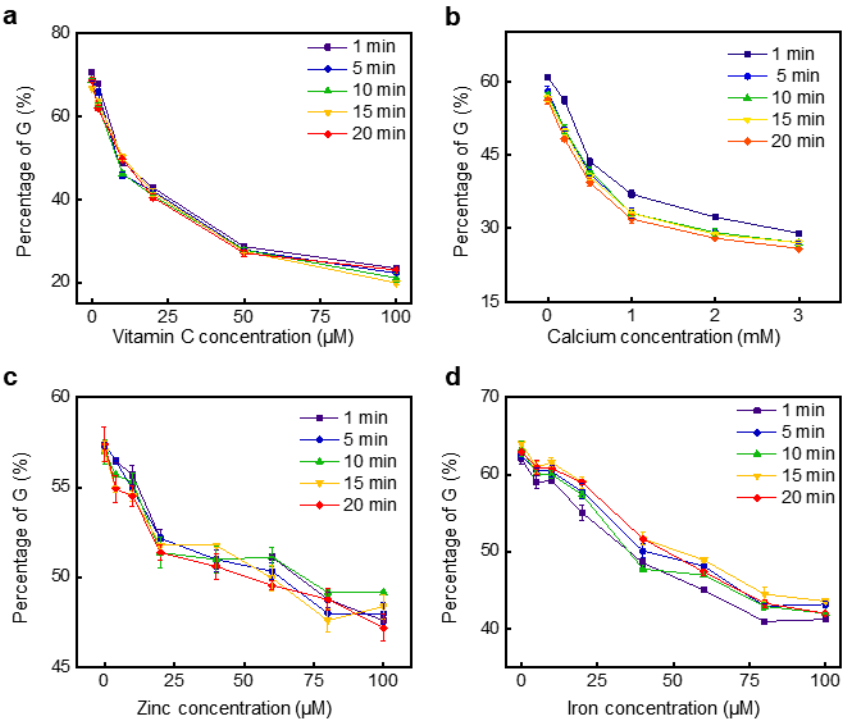

Figure S4. The normalized percentage of the green level captured at various times as a function of concentrations of **a,** vitamin C, **b,** calcium, **c,** zinc, and **d,** iron.

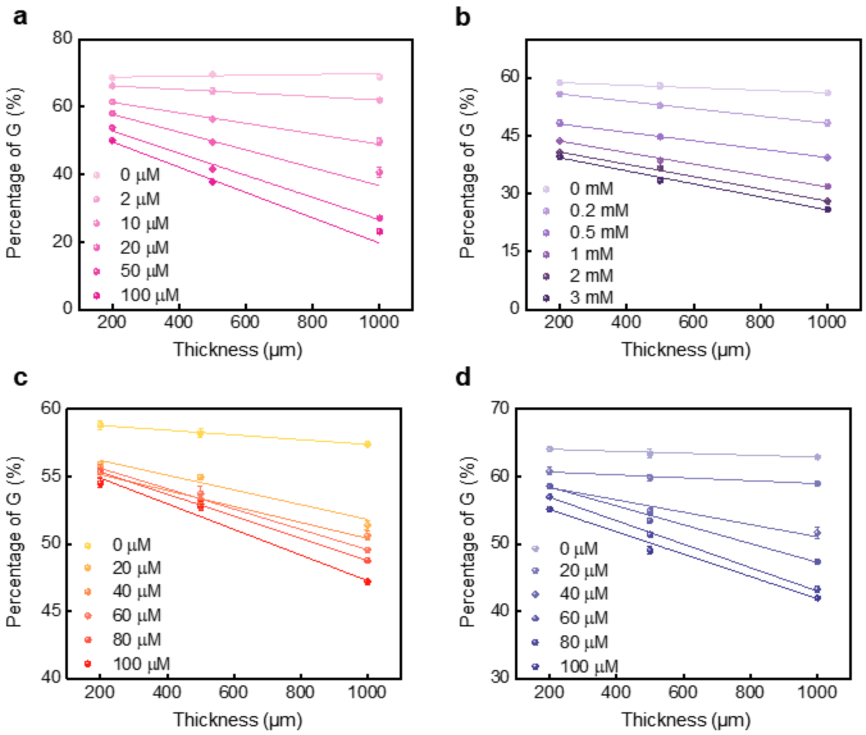

Figure S5. Normalized percentage of the green level as a function of thickness at various concentrations of **a,** vitamin C, **b,** calcium, **c,** zinc, and **d,** iron.

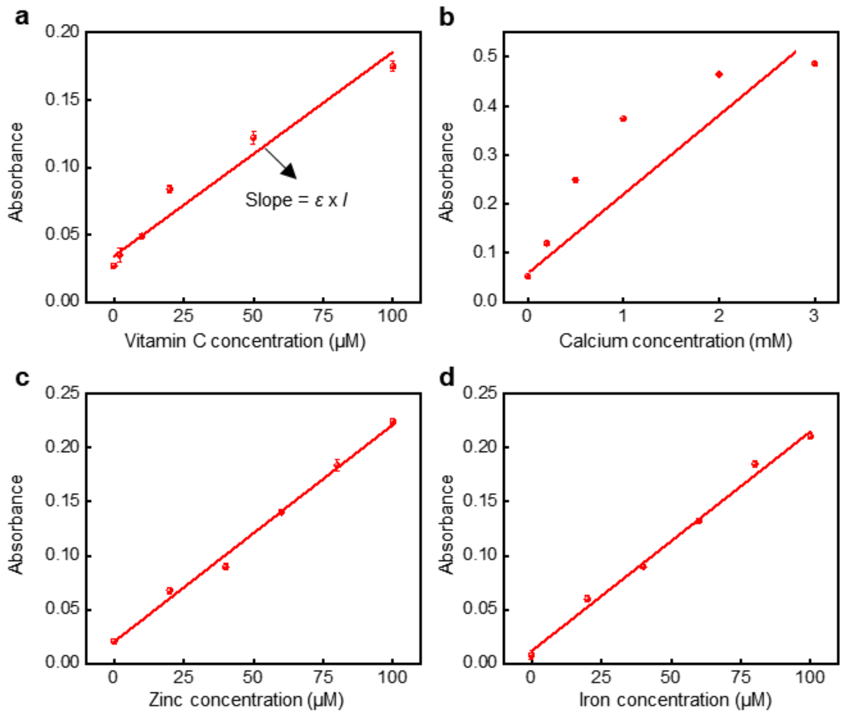

Figure S6. Calibration curve that relates the absorbance to the concentration for **a,** vitamin C, **b,** calcium, **c,** zinc, and **d,** iron.

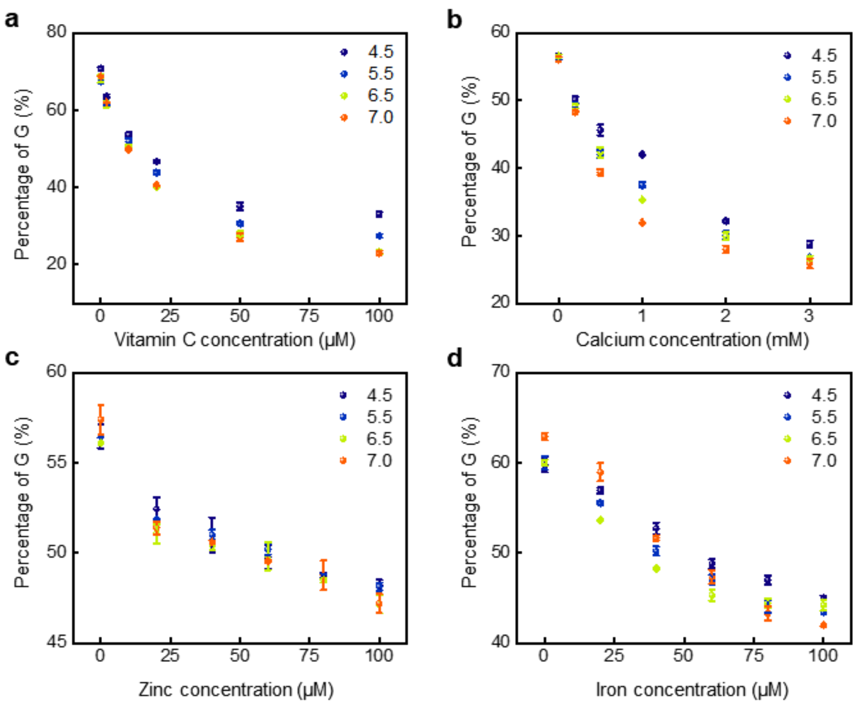

Figure S7. Normalized percentage of the green level at various pH as a function of concentration of **a,** vitamin C, **b,** calcium, **c,** zinc, and **d,** iron.

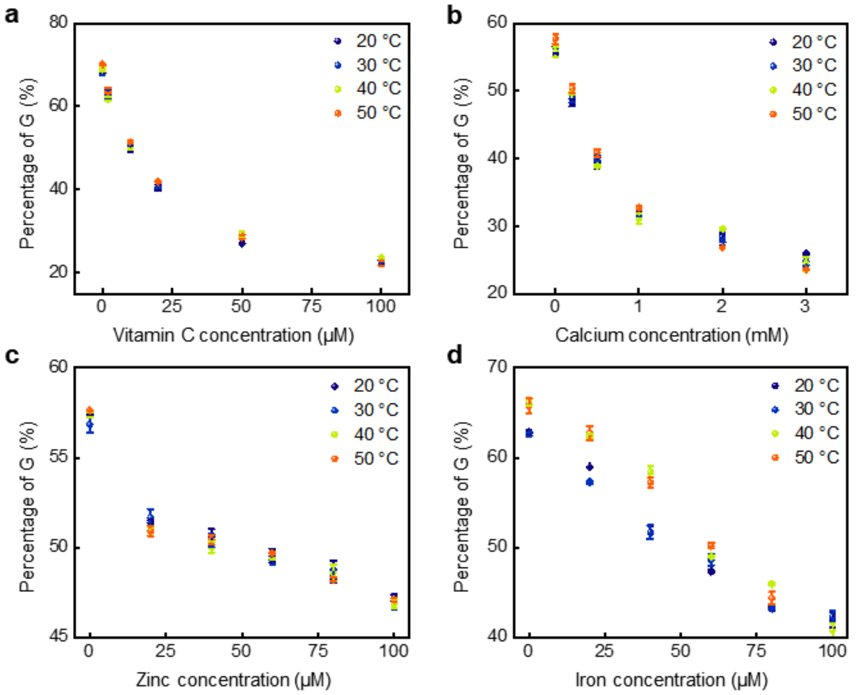

Figure S8. The normalized percentage of the green level at various temperatures as a function of concentration of **a,** vitamin C, **b,** calcium, **c,** zinc, and **d,** iron.

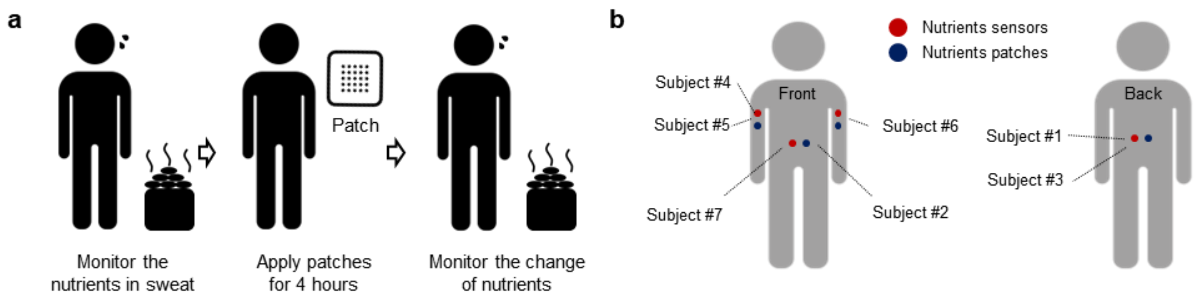

Figure S9. a, Schematic illustration of the nutrients sensor experiment before and after applying the vitamin patch for 4 hours. **b,** Illustration of the mounting locations of the sweat patches.

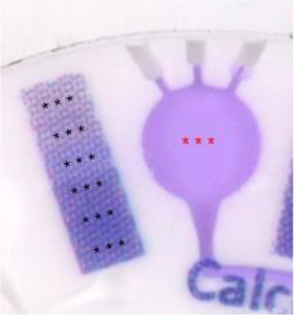

Concentration (mM)	Percentage of G value (%)
0	66.79
0.2	60.65
0.5	54.50
1	43.13
2	38.43
3	36.86
Reservoir	61.31

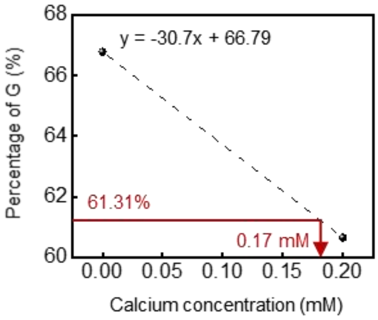

Figure S10. Interpolation method to analyze the concentration from the normalized color value of sweat filled reservoirs using prepared color reference markers.

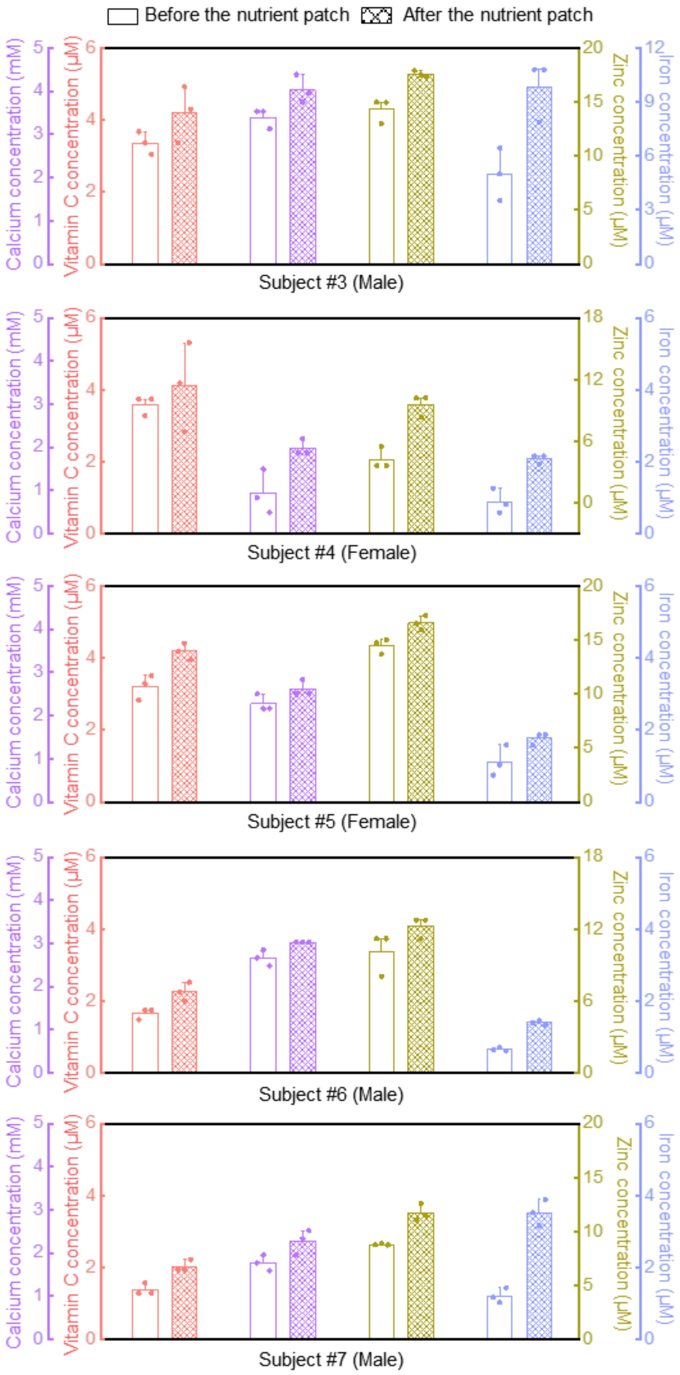

Figure S11. Graphs of the concentrations of nutrients in sweat before and after applying a nutrients patch for 4 hours from subject #3-#7.

	**Vitamin C (mg)**	**Calcium (mg)**	**Zinc (mg)**	**Iron (mg)**
**Vitamin water**	135	80	0	0
**Orange juice**	63	20	0	0
**Cereal**	7.2	80	1.65	10.8

Table S1. The nutrients content in vitamin water, orange juice, and cereal.
